# A Novel Strategy for the Synthesis of Amphiphilic and Thermoresponsive Poly(N-isopropylacrylamide)-*b*-Polystyrene Block Copolymers via ATRP

**DOI:** 10.3390/polym11091484

**Published:** 2019-09-11

**Authors:** Magdalena Fedorczyk, Anna Krzywicka, Piotr Cieciórski, Jan Romański, Elżbieta Megiel

**Affiliations:** Faculty of Chemistry, University of Warsaw, Pasteura 1, 02-093 Warsaw, Poland; magdalenafedorczyk1992@gmail.com (M.F.); a.krzywicka2@student.uw.edu.pl (A.K.); p.cieciorski@student.uw.edu.pl (P.C.); jarom@chem.uw.edu.pl (J.R.)

**Keywords:** smart materials, quasiliving polymerization, controlled radical polymerization, ATRP, thermoresponsive polymers

## Abstract

A new synthetic approach is presented for the preparation of Poly(N-isopropylacrylamide-block-styrene) PNIPAM-*b*-PS via an Atom Transfer Radical Polymerization (ATRP) technique. The proposed method is based on application of 2-chloro-N-(2-hydroxyethyl)propanamide (NCPAE) as a bifunctional initiator, which enables ATRP of two monomers, differing in activity and polarity, into two stages. The synthesized copolymer molecules contain two well-defined polymer chains connected by a linker, which is a derivative of the proposed initiator. Using NCPAE led to PNIPAMs with well-planned molecular weight, low polydispersities (PDI=1.1÷1.3) and hydroxyl functionality. Activation of such blocks for initiation of styrene polymerization was performed using α-bromoisobutyryl bromide. After such a modification, the synthesized homopolymers acted as macroinitiators in ARGET ATRP and a well-defined polystyrene block, as the next one in the polymer chain was successfully formed. Both of the synthesized macromolecules, PNIPAM and PNIPAM-*b*-PS, exhibit a thermoresponsive behavior with explicit lower critical solution temperatures (LCST) in their aqueous solutions. The synthesized homopolymers and subsequently derived block copolymers were characterized using Size-Exclusion Chromatography, Differential Scanning Calorimetry, Dynamic Light Scattering, and NMR spectroscopy.

## 1. Introduction

In the last two decades, Quasi-Living Radical Polymerization (QLRP) [1] has become a technique commonly used for the preparation of well-defined (co)polymers with tailor-made and advanced architectures; with such block copolymers and advanced nanostructures based on them [2]. Among QLRP techniques, Nitroxide Mediated Radical Polymerization (NMRP) [3,4], Reversible Addition-Fragmentation Chain Transfer Polymerization (RAFT) [5], and Atom Transfer Radical Polymerization (ATRP) [6] are the most commonly used. The last of them is especially useful because it enables quasiliving radical polymerization of a wide range of monomers, can be performed at relatively low temperatures (including room temperature), and leads to (co)polymers with narrow molecular weight distribution (MWD), precisely planned architectures, high functionalities and defined end-groups. In addition, recently developed modifications of ATRP such as Activators Generated by Electron Transfer Atom Transfer Radical Polymerization (AGET ATRP), Activators Regenerated by Electron Transfer Atom Transfer Radical Polymerization (ARGET ATRP) and Initiation for Continuous Activators Regeneration Atom Transfer Radical Polymerization (ICAR ATRP) allow control of radical polymerization using catalytic systems with concentrations at the ppm level [7].

It is worth noting that for the QLRP, a term controlled radical polymerization is commonly used. However, in fact the term controlled polymerization is not well-defined in scientific publications. It seems that from a mechanistic point of view, quasi-living polymerization is a better expression. Iván and Kennedy in several papers analyzed the distinctions between ideal living polymerization and quasiliving polymerization and clearly showed that using the expression “controlled” might be imprecise [8,9,10].

Recently, QLRP has been intensively used for the preparation of smart polymer materials, i.e., materials that have the ability to respond to external stimuli such as temperature, pH, electric and magnetic field, ionic strength, radiation forces and others [11,12]. This class of materials is extremely promising for a wide range of applications in the field of medicine, pharmacy, engineering, nanoscience, biochemistry and biotechnology [12]. Smart polymers can be successfully used as drug delivery agents, actuators, smart surfaces for tissue engineering, smart membranes with controlled porosity and selectivity, chemical valves, biosensor smart devices, artificial pumps, muscles and many others [11]. Among the smart polymers, temperature-responsive (co)polymers are some of the most intensively studied due to their unique property, which relies on the change of their solubility resulting from changing from a hydrophilic state to a hydrophobic state above a lower critical solution temperature (LCST) or below upper critical solution temperature (UCST) [12]. On the microscopic scale, it is visible as precipitate formation from a homogeneous solution of polymer; in the case of cross-linked hydrogels, prepared from smart polymers, as changes in their size connected with water content (hydrogel) [13,14]. For both LCST and UCST, a one-name phase transition temperature (PTT) is also used.

Poly(N-isopropylacrylamide) (PNIPAM) is a biocompatible, synthetic polymer which exhibits a LCST in aqueous solutions around 32 °C, importantly, close to the human body temperature. Below the LCST this polymer is completely miscible with water, in all proportions, whereas at temperatures above the LCST, a phase separation occurs. Importantly, this phenomenon is reversible and can be triggered externally. Therefore, PNIPAM is intensively studied in view of its potential applications, mainly in targeted drug delivery [15,16]. For these purposes, the LCST of the polymer must be appropriately adjusted. The LCST strongly depends on the molecular weight of the polymer and presence of co-monomers in the polymer chain [13]. Thus, adjustment of the LCST can be achieved, among other ways, by preparing a polymer with well-defined molecular weight followed by copolymerization with a second monomer differing in polarity. For this purpose, ATRP, as one of the most versatile QLRP techniques, can be successfully used. In such a case, choosing the initiator for the ATRP is one the most crucial issues for achieving successful process control. Since contrary to the classical free-radical polymerization, in the ATRP, the initiator takes part in an initiation process and its molecules are not only a source of initiating radicals but also a source of terminal atoms (bromine or chlorine radicals), which reversibly combine with a growing polymer chain. The terminal groups migrate between the active and dormant form of polymer chain, determining in this way the rate of polymerization [7]. In the case of N-isopropylacrylamid (NIPAM), which is a very active monomer, the employment of chlorine derivatives, as initiators usually provides good control under the conditions of ATRP. However, for many hydrophobic and less active monomers like a styrene, by applying bromine derivatives, as the initiators, significantly better results might be obtained in comparison with chlorine derivatives. This is because, the bond between the polymer chain terminated by styrene and chlorine is too strong; thus the migration of terminal group between the metal complex and active polymer chain is very slow, if at all possible.

So far, copolymers with the sequence PNIPAM-*b*-PS have been synthesized by using RAFT polymerization and the self-assembling of such copolymers into micelles has been studied [17,18]. This type of quasiliving polymerization has been used for preparation of the same blocks in reverse sequence, namely PS-*b*-PNIPAM [19,20]. In the case of RAFT polymerization, the prepared (co)polymers are terminated by dithiocarboxyl groups (S=C(Z)S–) which are able to interact with bioactive compounds, like proteins, therefore their biocompatibility can be limited.

ATRP has been employed for the preparation of PNIPAM-*b*-PS brushes onto the surface of silicon [21]. To this end, the silicon surface was functionalized with ethyl 2-bromoisobutyrate. Subsequently the ATRP of NIPAM and next styrene were carried out. Bromine derivatives were used as initiators in both polymerization steps. As discussed earlier, in the case of NIPAM polymerization by using chlorine derivatives, better results might be attained. Unfortunately, the copolymers attached to the surface were not analyzed in regard of their polydispersities. Importantly, as it turned out, the silicon wafers covered by PNIPAM-*b*-PS, which were obtained via the ATRP technique, are biocompatible. At the same time, such surfaces are thermosensitive which makes them very promising for biomedical applications.

In this paper, we report a new synthetic approach for the block copolymers of PNIPAM-*b*-PS via the ATRP technique. The proposed method relies on application of 2-chloro-N-(2-hydroxyethyl)propanamide (NCPAE) acting as bifunctional initiator. Functional groups in NCPAE opens up the opportunity to perform ATRP of two monomers differing in activity in a sequential manner. Based on this protocol, copolymers with molecules containing two well-defined polymer chains linked by the proposed initiator were synthesized. To the best of our knowledge, NCPAE has not been used as an initiator in ATRP; and the synthesis of block copolymers via the ATRP technique with a sequence wherein PNIPAM is the first block and PS is the second one has not been reported yet. Importantly, the developed synthetic route allows to build macromolecules with tailor-made length of hydrophobic block. Due to the sequence of the blocks, the synthesized molecules have an active end group on the hydrophobic side. Thanks this, a hydrophobic block can be easily extended, modified and attached to the surface. The last possibility makes the synthesized materials especially promising for the preparation of modified surfaces with hydrophobic inside layer and hydrophilic as well as thermosensitive outside layer. Such surfaces are highly desirable in tissue engineering and construction of smart membranes.

## 2. Experimental Section

### 2.1. Materials and Methods 

Solvents: dichloromethane (Avantor , Gliwice, Poland, 99%) was extracted with ultrapure water, rinsed with 5% aqueous solution of Na_2_CO_3_, saturated solution of NaCl, dried by using CaCl_2_ and afterwards fractionally distilled, N,N-dimethylformamide (DMF, Sigma-Aldrich, Steinheim, Germany, anhydrous 99.8%), 2-propanol (Avantor, Gliwice, Poland, 99.7%), were dried over molecular sieves (3A), chloroform (Avantor, Gliwice, Poland, 98.5%), methanol (Sigma-Aldrich, Steinheim, Germany, 99.8%), tetrahydrofuran (THF, Avantor, Gliwice, Poland, 99.5%), anhydrous tetrahydrofuran (Sigma- Aldrich, Steinheim, Germany, 99,9%), n-hexane (Avantor, Gliwice, Poland, > 90%), ethyl acetate (Avantor, Gliwice, Poland 99.5%) were used as received. Reagents. N-isopropylacrylamide (NIPAM, Alfa Aesar, Kandel, Germany, 97%) was recrystallized twice from hexane, styrene (St, Sigma-Aldrich, Steinheim, Germany) was dried using magnesium sulphate and next passed through a column filled with a basic alumina oxide (Sigma-Aldrich, Buchs, Switzerland, 98%, Brockmann activity I), CuCl_2_ (Sigma-Aldrich, Shanghai, China, 97%) was dried in vacuum oven at 120 °C for 24 hours. CuCl (Sigma-Aldrich, Shanghai, China, 97%) was rinsed with aqueous solution of SO_2_, next with a glacial acetic acid and finally with THF and dried in vacuum oven at 60 °C for 24 h, monoethanolamine (Sigma-Aldrich, Steinheim, Germany, 98%), triethylamine (TEA, Sigma-Aldrich, Steinheim, Germany, 99%), 2-chloropropionyl chloride (Sigma-Aldrich, Steinheim, Germany, 97%), CuBr (Sigma-Aldrich, Shanghai, China, 97%), CuBr_2_ (Sigma-Aldrich, Shanghai, China, 99%), tris[2-(dimethylamino)ethyl]amine (Me_6_TREN Sigma- Aldrich, Steinheim, Germany, 97%), N,N,N′,N″,N″-pentamethyldiethylenetriamine (PMDETA, Alfa Aesar, Kandel, Germany, 98%), 2,2′-bipyridyl (Sigma-Aldrich, Steinheim, Germany, 99%), α-bromoisobutyryl bromide (Sigma-Aldrich, Steinheim, Germany, 98%), tributyltin hydride (R_3_SnH, Alfa Aesar, Kandel, Germany, 97%), Tin(II) 2-ethylhexanoate (Sn(EH)_2_, Alfa Aesar, Karlsruhe, Germany, 96%) were used as received. Milli-Q ultrapure water (Merck KGaA, Darmstadt, Germany, resistivity 18.2 MΩ cm^−1^) was used throughout the experiments.

### 2.2. Analytical Method

#### 2.2.1. NMR Spectroscopy

^1^H and ^13^C NMR spectra were recorded on a Bruker Corporation 300 MHz or 500 MHz spectrometer. ^1^H NMR chemical shifts δ are reported in ppm referenced to tetramethylsilane (in CDCl_3_).

#### 2.2.2. Size-Exclusion Chromatography (SEC)

The analyses were carried out using Waters Alliance 2695 liquid chromatograph equipped with an RID detector (Waters Corporation 2414 RI). The separations of PNIPAMs were performed in 1% (*w/v*) LiBr/N,N-dimethylformamide (DMF), the copolymers PNIPAM-*b*-PS in tetrahydrofuran (THF) on three columns placed in series: Waters Styragel HR1, HR2, HR4 7,8 × 300 mm; The columns were thermostated at 35 °C (THF) and 25 °C (DMF). HPLC grade THF (AppliChem) was used at a flow rate of 0.8 mL min^−1^ and DMF p.a. (Sigma-Aldrich, Steinheim, Germany) at a flow rate of 1 mL min^−1^. The concentration of samples prepared in eluents was 2 mg mL^−1^, 20 µl injections were applied. PS standards were used for calibration (Shodex) in the range 1.31 × 10^3^ to 3.64 × 10^6^ Da.

#### 2.2.3. Differential Scanning Calorymetry (DSC)

Critical solution temperatures (CSTs) and corresponding enthalpies were determined on the basis of DSC heating thermograms recorded for aqueous solutions of (co)polymers with different concentrations (in the range 0.1–3% *w/w*). Hermetic aluminum crimp-seal pans (50 μl) were used. DSC measurements were performed using a TA Instruments DSC Q20 calorimeter with thermobalance (precision ±0.4%; minimal mass 0.02 mg) under an N_2_ atmosphere with scanning rate 1 K min^−1^. The enthalpies of the phase transition process were determined as an area of the measured endothermic peak. Pure indium was used for temperature and enthalpy calibration of the DSC module. The glass transition temperatures (*T*_g_) were determined as the temperature corresponding to half the complete change in heat capacity in the cooling cycle of the DSC scan at a rate of 20 K min^−1^. First the samples were heated from 20 °C to 150 °C at a rate of 10 K min^−1^. Next they were kept at 150 °C for 5 min. to eliminate the thermal history. Finally, they were cooled to 20 °C at a rate of 20 K min^−1^.

#### 2.2.4. Dynamic Light Scattering (DLS)

Dynamic Light Scattering (DLS) measurements were performed with a Zetasizer Nano series apparatus (Malvern) with a laser He-Ne (4 mW) at 632.8 nm and a thermostatted cell holder. Hydrodynamic diameters of particles present in the aqueous solutions were measured at 25, 35 and 45 °C. The solutions were equilibrated for 10 min before the measurements.

#### 2.2.5. Gas Chromatography Analysis (GC)

GC was used to determine conversion of NIPAM and styrene conversion (in the second stage) using an Agilent 7820A gas chromatograph equipped with an FID detector with 1,4-dioxane or DMF as an internal standard. Analysis was carried out first isothermally at 50 °C for 6 min and next the temperature was increased to 150 °C with a heating rate of 300/min. Conversion of the monomers was determined as the decrease of the monomer peak area relative to the area of the standard’s peak (1,4-dioxane for NIPAM and DMF for styrene analysis, respectively).

#### 2.2.6. Elemental Analysis (EA)

The analysis of Br concentration in the PNIPAM-Br was done using the Schöninger flask combustion method. The sample was combusted in an oxygen-enriched environment in the presence of a Pt catalyst. The resultant gases were absorbed and a titration with Hg(ClO_4_)_2_ was performed with an ethanolic solution of diphenylcarbazone, as an internal indicator.

### 2.3. Synthetic Procedures

#### 2.3.1. Synthesis of 2-Chloro-N-(2-Hydroxyethyl) Propanamide (NCPAE)

A round bottom flask (250 mL) with a stirred solution of ethanoloamine (2.5 mL, 41.4 mmol) and triethylamine (2 mL, 14.3 mmol) in dry methylene chloride was immersed in an ice bath (0°C) under an argon atmosphere. Then 2-chloropropionyl chloride (1 mL, 10.3 mmol) in 20 mL of dry dichloromethane was added dropwise over 1 h. After addition was complete, the ice bath was removed and the reaction mixture was stirred overnight at room temperature. After this time, the mixture was evaporated and the residue was separated by column chromatography using 5% methanol in chloroform as an eluent. The product was obtained in the form of colorless oil and was crystallized from an ethyl acetate: hexane (1:1) mixture to give a white solid (1.43 g, 9.2 mmol) in 92% yield.

HRMS (ESI): calcd for C_5_H_11_ClNO_2_ [M+H]^+^: 152.0473, found: 152.0431. HRMS (ESI): calcd for C_5_H_9_ClNO_2_ [M–H]^−^: 150.0327, found: 150.0368. ^1^H NMR 300 MHz, 7.04 (bs, 1H) –NH–, 4.42 (q, J = 7, 1H) –CH(Cl)CH_3_, 3.80–3.70 (m, 2H) –CH_2_–OH, 3.50–3.35 (m, 2H) –NH–CH_2_–, 2.65 (t, J = 6, 1H) –OH, 1.73 (d, J = 9, 3H) –CH_3_; ^13^C NMR (75 MHz, CDCl_3_) δ 170.6 C=O, 61.9 –CH_2_–OH, 55.9 –CH(Cl)CH_3_, 42.6 –NH–CH_2_–, 22.7 –CH_3_; mp. 67–69 °C.

#### 2.3.2. Polymerization of NIPAM with NCPAE as an Initiator

The polymerization of NIPAM was carried out using the procedure described by Xia et al. [22]. with several modifications—namely, another initiator was used in our procedure and also the method for preparing the polymerization mixture was changed.

The molar ratios of all reagents used in the polymerizations are given in Table 1.

In a typical experiment, NIPAM and CuCl were added to a dried and degassed Schlenk flask sealed with a silicone rubber septum and equipped with a stir bar. The flask was degassed using a Schlenk line and filled with a dry argon. Next, dry isopropanol (half of the total volume used in the polymerization) was transferred into the flask and the mixture was bubbled with argon for 20 min. Afterwards, an appropriate volume of Me_6_TREN was injected into the flask using a syringe. After addition of the ligand the mixture immediately turned from colorless to light yellow. The mixture was stirred and bubbled with argon for the next 20 min and after this time a solution of NCPAE in isopropanol (earlier degassed by bubbling with argon) was injected to the flask using a syringe (in counter flow of argon). The mixture was left under an argon atmosphere (under slight positive pressure) on a magnetic stirrer at room temperature for the appropriate periods of time (from 2 to 23 h) and terminated by blowing air through the flask. To determine the NIPAM conversion, during the polymerization time, samples were taken periodically under argon using an argon-purged syringe and diluted with acetone; and were subsequently analyzed using gas chromatography (GC).

As a result of the polymerization, a viscous, green, and homogeneous mixture was obtained. The polymerization was stopped after an appropriate period of time (see Table 1) by opening the flask and blowing air through the flask using a syringe. After the polymerization, the mixture was dissolved in THF, concentrated using vacuum rotary evaporator and next passed through a basic alumina column to remove the catalyst system. The polymers were isolated by precipitation with hexane. Finally, the white solid products were dried under vacuum for 48 h at 60 °C. The long polymer drying time ensured that the monomer was completely removed (NIPAM sublimes easily at elevated temperature). The monomer conversion was determined gravimetrically and also using GC analyses. ^1^H NMR spectra proved that the polymers didn’t contain a residual of monomer.

^1^H NMR: 300 MHz, CDCl_3_ δ: 6.75–5.80 (bs) NH, 4.01 (bs) –CH(CH_3_)_2_, 2.30–1.25 (bs) –CH_2_–CH(R)–, 1.14 (bs) –CH(CH_3_)_2_.

#### 2.3.3. Dehalogenation of PNIPAM (Removal of Terminal Chlorine Atoms)

The dehalogenation of PNIPAMs was carried out using the procedure described by Coessens and Matyjaszewski [23].

In brief, PNIPAM and CuBr were added to a dried Schlenk flask sealed with a silicone rubber septum and equipped with a stir bar. The flask was degassed using a Schlenk line and filled with a dry argon. Next, a dry THF was transferred into the flask and the mixture was bubbled with argon for 10 min. Afterwards an appropriate volume of PMDETA was injected into the flask using a syringe. The mixture turned intensively green after the addition of PMDETA. For the next 30 min, the mixture was stirred and bubbled with argon. Then, tributyltin hydride (R_3_SnH) was injected to the flask using a syringe (in counter flow of argon). The same molar ratio was used in all experiments PNIPAM:CuBr:PMDETA: R_3_SnH equal to 1:0.5:0.5:3. The flask with the reaction mixture was immersed in a thermostatically controlled oil bath heated at 60 °C with constant stirring (using magnetic stirrer) for 4 h. During the reaction, the color of the mixture changed from green through brown to dark brown almost black. The reaction was stopped by opening the flask and introducing air into it using a syringe. As a result of contact between the post-reaction mixture and air, the original green color of the mixture appeared again. Next, the post-reaction mixture was passed through a basic alumina column to remove the catalytic system. Afterwards, the solution was concentrated using a vacuum rotary evaporator; THF was used to dissolve the residue; and the polymers were isolated by precipitation with hexane (1:5 THF:hexane *v/v*). Finally, the white solid products were dried under vacuum for 24 h at 60 °C.

#### 2.3.4. Esterification of Functionalized PNIPAMs with α-Bromoisobutyryl Bromide (the Preparation of PNIPAM-Br)

The PNIPAM was added to a dried Schlenk flask sealed with a silicone rubber septum and equipped with a stir bar. The flask was degassed using a Schlenk line and filled with dry argon. Next, a dry THF was transferred into the flask (40 mL/1 g of PNIPAM), and TEA was injected to the flask using a syringe. Then the solution was bubbled with argon for 10 min. Afterwards, the degassed solution of α-bromoisobutyryl bromide in anhydrous THF was injected into the flask using a syringe (in counter flow of argon) and the reaction mixture was stirred at room temperature. for 24 h. During the reaction, a small amount of light-yellow solid was precipitated. The precipitated solid (most likely trimethylamine hydrobromide) was removed from the post-reaction solution using a syringe filter (0.45 μm). The polymers were isolated by precipitation with hexane, filtered and dried under vacuum for 24 h at 60 °C. Elemental analysis showed the content of bromine in the synthesized polymers in the range 1–2.3%.

#### 2.3.5. Chain Extension of the PNIPAM-Br Macroinitiator with Styrene

The PNIPAM-Br was added to a dried Schlenk flask, sealed with a silicone rubber septum and equipped with a stir bar. Next, it was dissolved in a mixture of DMF and styrene (St) (1:1 *v/v*). The obtained solution was bubbled with argon for 20 min. A solution of ligand (PMDETA, Me_6_TREN or bpyr) with Cu(II) salt in DMF (earlier degassed by bubbling with argon) was injected into the flask using a syringe (in a counter flow of argon). The solution obtained in this way (almost colorless) was bubbled with argon for the next 20 min and stirred using a magnetic stirrer. Afterwards, tin(II) 2-ethylhexanoate (Sn(EH)_2_) was injected to the flask using a syringe (in counter flow of argon). The same molar ratio of all reagents was used in all experiments PNIPAM:St:Cu(II):PMDETA/Me_6_TREN/bpy:Sn(EH)_2_ 1:400:0.006:0.1:0.1. The flask with reaction mixture was immersed in a thermostatically controlled oil bath heated at a set temperature, with constant stirring (using a magnetic stirrer) for an appropriate period of time (see Table 2). The copolymerization was carried out for a period of time in the range from 4 h to 46 h (see Table 2). The copolymerization was stopped, after an appropriate period of time, by blowing air through the flask. Next, the mixture was evaporated to dryness and dissolved in THF. The copolymers were isolated by precipitation with hexane and subsequently filtered, washed with toluene and dried under vacuum for 24 h at 60 °C. The filtrates obtained after separation of copolymers were concentrated using a rotary vacuum evaporator and methanol was added in the volume ratio hexane:methanol 5:1. The precipitated white solids were filtered and dried under vacuum for 24 h at 60 °C.

^1^H NMR: 300 MHz, CDCl_3_ δ: 7.25–6.80, 6.75–6.25 (bs) NH (PNIPAM block) and ArH (PS block), 4.01 (bs) CH(CH_3_)_2_ (PNIPAM block), 2.30–1.25 aliphatic fragments –CH_2_–CH(R) (PNIPAM and PS blocks), 1.14 (bs) CH(CH_3_)_2_ (PNIPAM block).

## 3. Results and Discussion

**Scheme 1:**
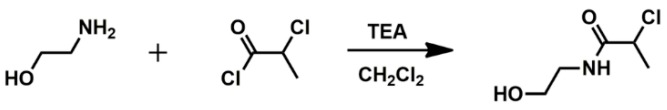
The scheme of synthesis of the bifunctional initiator 2-chloro-N-(2-hydroxyethyl)propanamide (NCPAE).

NCPAE was synthesized as a result of acylation of ethanoloamine by 2-chloropropionyl chloride (Scheme 1).

**Figure 1:**
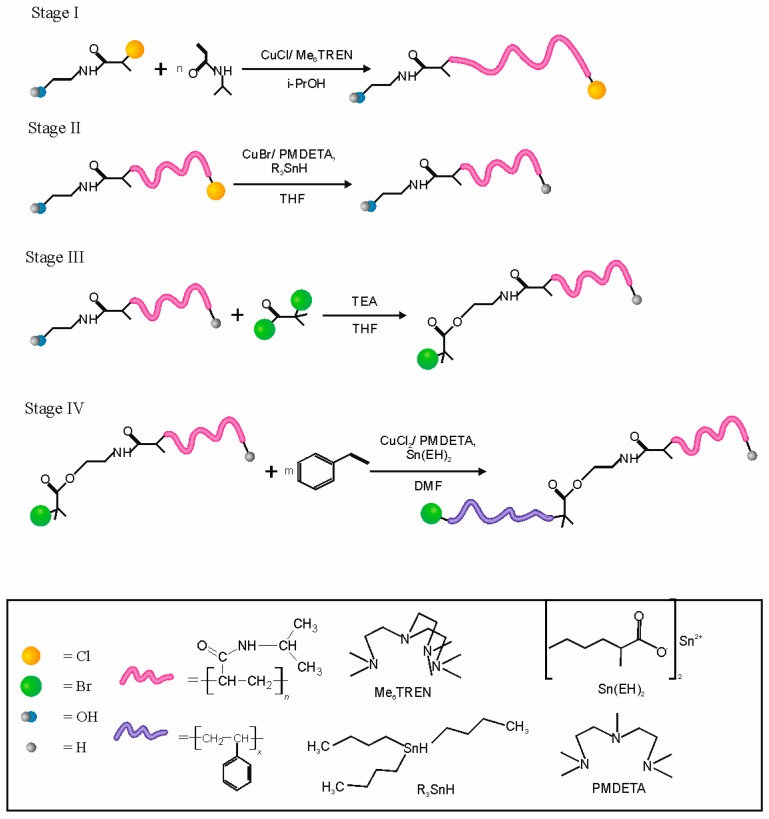
The scheme of the procedure leading to the preparation of PNIPAM-block-PS copolymers using the bifunctional initiator 2-chloro-N-(2-hydroxyethyl)propanamide (NCPAE).

The synthesized NCPAE was used as an initiator for polymerization of N-isopropylacrylamide (NIPAM) via the ATRP mechanism (Figure 1, Stage I). Due to the presence of chlorine atoms in the molecule of NCPAE it can be used as an initiator of ATRP for such active monomers as NIPAM. On the other hand, application of NCPAE as an initiator enables synthesis of chlorine atom-terminated polymers on one side and hydroxyl groups on the opposite side of the molecule. Our earlier investigations proved that practically it is not possible to achieve a chain extension with styrene when PNIPAM is chlorine terminated (Table S1 in Supplementary Material).

In such a macroinitiator (PNIPAM-Cl), the chemical bond formed between chlorine atoms and the growing polymer chain terminated with styrene is probably so strong that a transfer of chlorine atom on to the catalytic system is not possible. Therefore, we decided to design such an initiator, which enables modification of the PNIPAM chain with bromine. The hydroxyl group in the polymers, prepared using the method developed by us, provides such a possibility.

However, before the modification of the polymers with bromine, the removal of terminal chlorine atoms was performed. This is because our earlier studies showed, that 2–8 (no more) of styrene mers can be attached to PNIPAM’s chains terminated with chlorine under ATRP conditions. This process may contribute to the deterioration of the polydispersity of the synthesized polymers.

After the dehalogenation process (Figure 1 Stage II), the next step was to functionalize the polymer chains using α-bromoisobutyryl bromide. As a result, PNIPAM-Br macroinitiators were synthesized that could be used in the polymerization of styrene (Figure 1, Stage IV). The developed procedure allowed the successful synthesis of amphiphilic and thermosensitive block copolymers with PNIPAM, as the first block and PS as the second one.

The synthesized (co)polymers were characterized using spectroscopic (^1^H NMR) and chromatographic (SEC) methods, thermal analyses (DSC) and dynamic light scattering method (DLS).

The molar ratios applied in the polymerization of NIPAM and characteristic of the obtained homopolymers from SEC analyses are presented in Table 1.

The polymerization of NIPAM was carried out using a procedure described in the literature [22] but with several modifications. First of all, we used an initiator which allows terminal functionalized polymers with hydroxyl groups to be obtained. It is worth pointing out that this is the first time such an initiator has been used in ATRP. Furthermore, we changed the method of preparation of the polymerization mixture before addition of the initiator. According to our procedure NIPAM, CuCl and half of the total volume of isopropanol used in the polymerization were placed directly in a reaction vessel. Next, the mixture was degassed; and then Me_6_TREN was introduced into the vessel. In this way, we could omit a step aimed at transferring the mixture with suspended CuCl (not dissolved in this mixture) to the reaction vessel, as required by the procedure of Xia et al. [22] We observed that this step in the procedure can lead to loss of some CuCl and may therefore be responsible for poorer polymerization control. The polymerization was carried out with molar ratios of initiator to monomer 1:50 and 1:200 and for periods of time from 2 h to 23 h (see Table 1). The obtained polymers were purified and analyzed by SEC. Table 1 presents the comparison of theoretical molecular weights for the obtained polymers (calculated using a molar ratio of initiator (I) to monomer (M) and obtained conversion (conv)) with the molecular weights determined for them from the SEC analyses as well as polydispersity indices. Most of the SEC analyses were carried out using 1% solution (*w/v*) LiBr/DMF as the mobile phase. This is because our earlier studies proved that, in the case of separation of hydrophilic polymer materials on a column packed with cross-linked polystyrene (PS)-divinylbenzene (DVB) copolymer particles, using THF as an eluent is difficult. Application of DMF with the addition of LiBr as an eluent allows lower retention of such by the PS-DVB stationary phase [24,25].

Application of NCPAE as an initiator and modification of the procedure reported by Stöver and co-workers [22] allowed very good control of NIPAM polymerization, and high functionality could be achieved. The molecular weights expected on the basis of the applied molar ratios M:I and the obtained conversions are very close to those obtained from the SEC analyses.

For the same molar ratio of NIPAM to methyl 2-chloropropionate, as an initiator, Xia et al. [22] reported higher conversions at for the same polymerization time and molecular weights from SEC almost twice as high as predicted theoretically. A similar situation was observed by these authors for higher [M]:[I] ratios, namely 100:1, 200:1 and 400:1. In our system, we observed the opposite situation for the higher ratio 200:1—the molecular weight determined from SEC analysis is smaller than the theoretical weight (sample P6). It is likely that both the application of the initiator proposed in this work and modification of the procedure caused the polymerization to run slower.

The polydispersity indices PDI (defined as the ratio of weight-average molecular mass and number average molecular mass *M*_w_/*M*_n_) determined from SEC analyses are relatively low, close to 1 (<1.2) for the polymers that were obtained using a molar ratio M:I = 50:1. In the case of the molar ratio 200:1, the polydipersity is insignificantly higher (1.31). Importantly, the application of NCPAE as an initiator in molar ratio [M]: [I] 50:1, together with modification of the procedure proposed by Stöver and co-workers, gave excellent agreement between theoretical and experimental *M*_n_ molecular weights (see Table 1).

However, when the molar ratio [M]: [I] = 200:1 was used, the experimental molecular weight from SEC (11,700 Da) was significantly lower than theoretical one (14,400 Da) and PDI index was higher (1.31).

**Figure 2:**
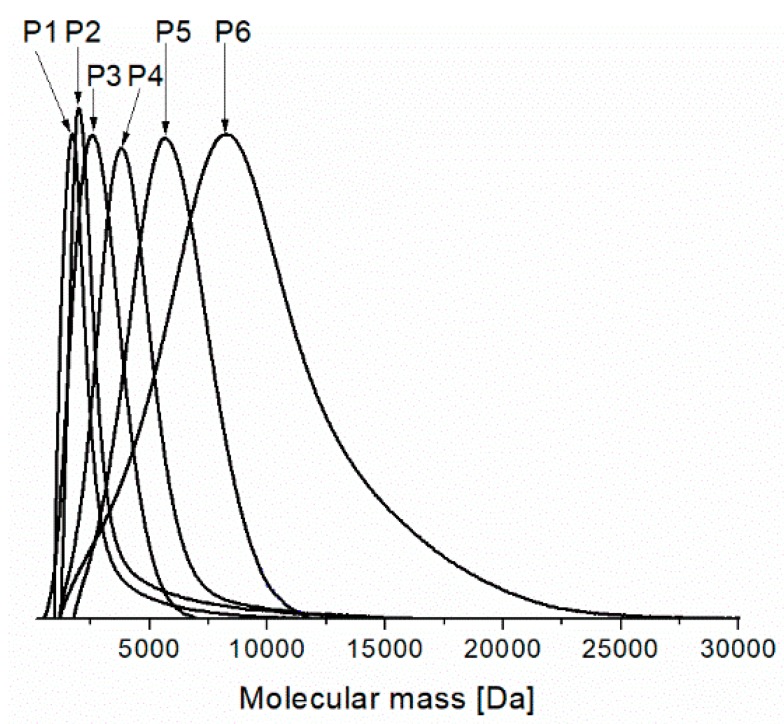
The molecular weight distribution curves from Size-Exclusion Chromatography (SEC) analyses for the prepared PNIPAMs (descriptions of the samples P1–P6 and polymerization conditions are given in Table 1).

Figure 2 depicts the molecular weight distribution from SEC analyses for all the prepared PNIPAMs. For P1–P5 the curves are very narrow whereas for P6 they are relatively narrow with tailing on higher molecular weight side. It indicates that fractions with significantly higher molecular weights are present in the samples and not all polymer chains were growing with the same rate but also termination processes can be responsible for the presence of polymer chains with high molecular weight in the polymerization system. Application of smaller amounts of the catalyst system (higher molar ratio of monomer to initiator) does not allow to obtain polymers with narrow MWDs. This drawback is probably connected with a partial deactivation of the catalyst system as a result of complex formation between the polymer amide groups and the copper salt. Such a complex exhibits a low catalytic activity. Teodorescu et al. proposed the formation of such a complex as the reason for the observed difficulties in ATRP of several acrylamides [26], Brittain made the same suggestion for ATRP of N,N-dimethylacrylamide [27].

**Figure 3:**
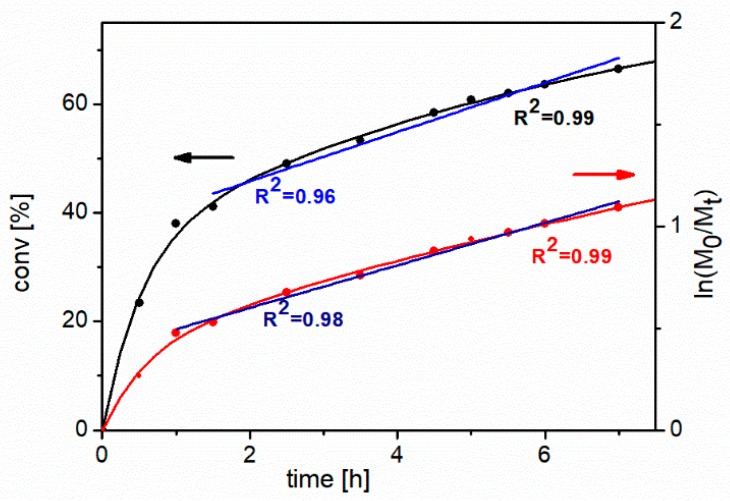
Conversion of monomer vs. time and first-order kinetic plot (right axis) for the Atom Transfer Radical Polymerization (ATRP) of NIPAM. Polymerization conditions: [NIPAM]:[NCPAE]:[CuCl]:[Me_6_TREN] = 50:1:1:1, room temperature, isopropanol as a solvent (1:2 NIPAM:solvent, *w/w*). The black and red arrows indicate a proper axis for the relationship shown in the same color.

To study the kinetics of the process of NIPAM’s polymerization, the monomer concentration was monitored using gas chromatography (GC) in short time intervals for 7 hours. Figure 3 shows the first-order kinetic plot and NIPAM’s conversion vs. time of polymerization. It is clearly visible that, during the first 2 hours, this process is very fast, and the first-order kinetic plot shows significant curvature. However, after 2 hours, the polymerization slows down and during the next 4 hours the relationship is almost linear with relatively good correlation coefficient (*R*^2^ = 0.96). In whole range of the polymerization time relationship, conversion vs. time can be described by an exponential function with a very good correlation coefficient (*R*^2^ = 0.99). In view of the obtained results it seems that the polymerization rate at the first period of the reaction should be slowed down. Probably decreasing of temperature and concentration of monomer should allow to achieve a linear kinetic in whole range of the polymerization time.

**Figure 4:**
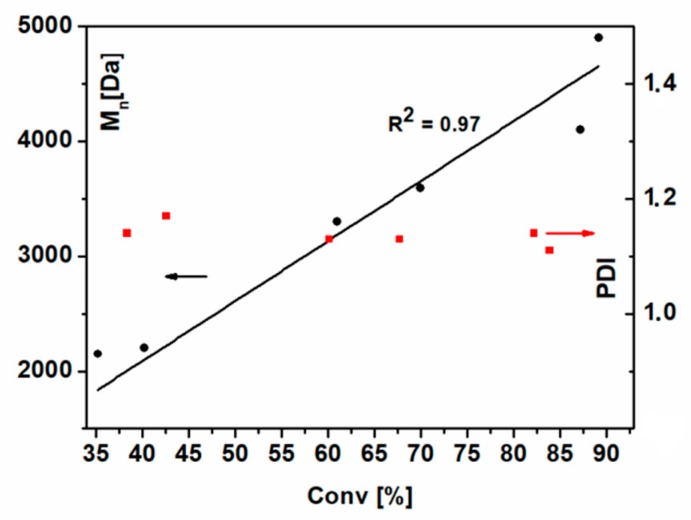
Number-averaged molecular weight and polydispersity indices vs. monomer conversion. Polymerization conditions: [NIPAM]:[NCPAE]:[CuCl]:[Me_6_TREN]=50:1:1:1, room temperature, isopropanol as a solvent (1:2 NIPAM:solvent, *w/w*). The black and red arrows indicate a proper axis for the relationship shown in the same color.

Figure 4 shows the dependence of the number-averaged molecular weight (*M*_n_) and polydispersity, determined from the SEC analyses, on the monomer’s conversion.

The linear relationship between *M*_n_ and conversion is fairly good (*R*^2^ = 0.97) and the PDI values are below 1.2 in the whole analyzed range of monomer conversions (from 30% to 90%). This indicates that the contribution of termination reactions is significantly diminished, even for high conversions.

The DSC and DLS corroborated that all prepared homopolymers clearly exhibit thermosensitivity with explicit LCST in their aqueous solutions. The critical solution temperatures (CSTs) determined from the DSC measurements are given in Table 2. The three temperatures have been determined: *t*_o_ as onset of the peak, *t*_p_ as the minimum of the peak and *t*_r_ as the range of temperatures within which the transition occurs (under the conditions of DSC measurements).

**Figure 5:**
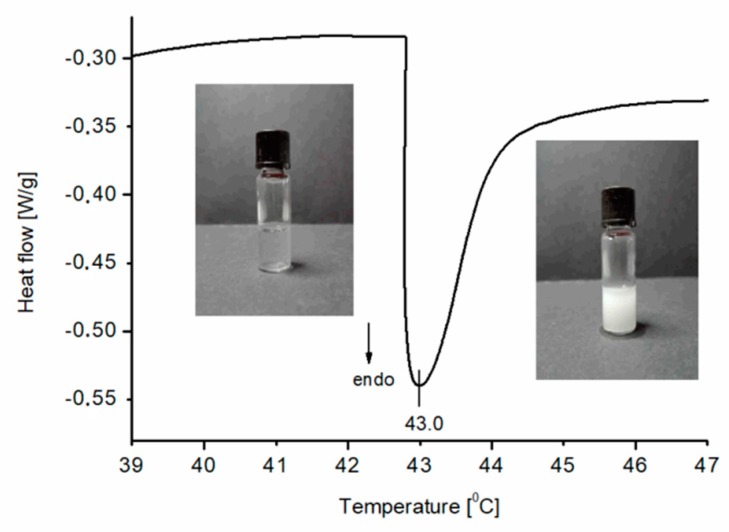
The heating DSC thermogram for an aqueous solution of P1 (1.5% *w/w*) and photos of the solution below and above the phase transition corresponding to the endothermic effect visible on the curve. The heating rate of 1 K/min.

Figure 5 depicts a typical heating DSC curve, recorded for an aqueous solution of the synthesized PNIPAM (sample P1) and also shows pictures of the solution which have been taken below and above the thermal phase separation corresponding to the visible endothermic peak. As can be seen in Table 2, thermal phase separation occurs at the lowest temperatures for polymer P6. For this polymer, the molecular weight determined from SEC is highest among those studied. However, the differences between other samples are insignificant and the peak temperature occurs around 43 °C. The lack of differences between the CST for the other samples might be a result of relatively small differences between their molecular masses (2–5 kDa).

On the other hand, the number-averaged molecular mass of P6 is not significantly higher but its polydispersity is significantly higher (PDI=1.31) and the presence of the fraction with higher molecular mass might be responsible for the decreased CST. The CSTs determined by DSC are very close to the values reported by Xia et al. [22] for the PNIPAMs with similar molecular weights and MWDs.

The phase transition enthalpies, estimated as areas of the corresponding endothermic peaks, are also presented in Table 2. The estimated enthalpies are within the range 3–14 J per 1g of solution with a concentration 1.5% (*w/w*) which corresponds to a concentration of 1.3 × 10^−4^ in repeating units (NIPAM mers) of mol per 1g of solution. These values are several times higher than those determined by Kano and Kokofuta [28] for solutions with similar concentration of PNIPAM. The differences in the determined values may result from the differences in size of polymer molecules and polymer dispersity. Kano and Kokofta performed DSC measurements for PNIPAM with molecular mass 5.5 × 10^2^ kDa prepared by classical radical polymerization, so with a wide MWD. Probably, in the case of polymers built of molecules with lower molecular weights, like those studied here, a transition from the coiled state to the globular conformation requires more energy. Most likely, more hydrogen bonds between short polymer chains and water molecules must be broken to change the conformational state.

The higher transition temperatures for our samples, in comparison to those determined by Kano et al. [28] also indicate that, in the case of PNIPAMs with smaller molecular mass and lower polydispersity, the phase separation requires more energy and therefore occurs at higher temperature. Nevertheless, we didn’t find any correlation between molecular mass and endothermic enthalpy (Δ*H*) of the thermal phase transition. The differences in molecular masses of the prepared polymers are probably too small to see any relationship between them; or such a relationship doesn’t exist. On the other hand, as it was recently reported, not only molecular weight has an influence but also the experimental conditions, such as concentration, the heating rate, wavelength of light in optical measurements, have also significant effect on the observed CST [29,30,31].

The dynamic light scattering (DLS) technique was also used to examine the thermosensitivities of the prepared polymers. Importantly, all polymer samples were dissolved in ultra-pure water at least 1 h before the measurements to give sufficient time for hydration sphere formation around the polymer molecules. 

**Figure 6:**
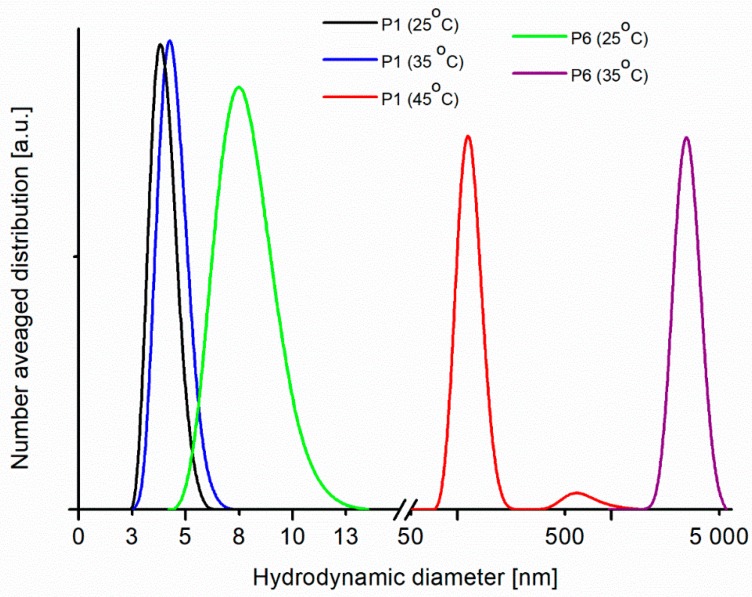
The number-averaged hydrodynamic diameter distributions in aqueous solutions of P1 and P6 at 25, 35 and 45 °C from Dynamic Light Scattering (DLS) measurements.

The hydrodynamic diameters of particles were determined in the solutions at 25, 35 and 45 °C. Figure 6 shows the number-averaged hydrodynamic diameter distributions in aqueous solutions of P1 and P6 with the same concentration (1.5% *w/w*).

As can be seen in Figure 6, an aggregation process occurs when the temperature increases to 45 °C in the solution of P1 and at 35 °C in solution of P6. Among the polymers studied, the P1 sample is the polymer with the lowest molecular weight, and for this sample at 35 °C only a marginal increase of particle hydrodynamic diameter can be observed. However, an aggregation process is evident at 45 °C. For the polymer P6, with six times bigger molecular weight, an aggregation occurs at lower temperature and only particles with diameter above 2,000 nm are visible in the solution at 35 °C. The results from DLS are consistent with the results from DSC measurements, namely for the polymers with lower molecular mass and lower polydispersity the phase transition occurs at higher temperature, which means that the LCST is for them higher. The results from DLS measurement are similar for other polymers (P2–P5) and the determined sizes of particles at temperatures below and above LCST are in the range between these obtained from measurements for P1 and P6 (see Figure S1 in the Supplementary Material).

The polymers prepared using NCPAE as an initiator and CuCl/Me_6_TREN as a catalyst are terminated by chlorine (see Figure 1, Stage I). Thus, theoretically it should be possible to use them as macroinitiators in ATRP of styrene. However, our studies proved that the chain extension with styrene of PNIPAMs terminated with chlorine (PNIPAM-Cl) occurs with a very poor initiation efficiency and only 2–8 mers could be attached (see Table S1 in Supplementary Material). Therefore, we decided to develop a procedure which replaces a terminal chlorine atom with a terminal bromine atom; although this replacement doesn’t necessarily occur can at the same position in the polymer molecule. To avoid a partial extension of the polymer chains via transfer of chlorine atoms, first the removal of chlorine atoms from the polymer chains was performed through a dehalogenation process. The dehalogenation was carried out using tributyltin hydride according to the procedure described by Coessens and Matyjaszewski [23]. After the dehalogenation process, only minimal changes in molecular weight and molecular weight distribution were observed (see Table S2 in the Supplementary Material).

Due to the presence of hydroxyl groups in the synthesized PNIPAMs they can be facilely modified. In the developed procedure we used α-bromoisobutyryl bromide as an acylation reagent and in this way, we introduced a bromine atom, which may be transferred using a catalytic system in ATRP (see Figure 1, Stage III). After the esterification the molecular masses and polydispersity indices of the obtained modified polymers were determined using SEC (see Table S2 in the Supplementary Material). Using elemental analysis and the number-averaged molecular mass of the polymers from SEC, the degree of functionalization of the hydroxyl groups in the polymer chain by bromine atom was determined and it was within the range 75–85%.

The synthesized polymers functionalized with bromine (PNIPAM-Br) were then used as macroinitiators in Activators Regenerated by Electron Transfer ARGET ATRP with styrene. We chose this type of ATRP for several reasons. First of all, the ARGET modification enables ATRP with a very low concentration (< 50 ppm) of the catalytic system. Thus, the synthesized copolymers practically can be used without a laborious procedure of purification from a catalyst. The purity of the polymers is crucial, in particular for their medical applications. The amount of catalyst may be significantly reduced in this technique due to the employment of a reducing agent such as tin(II) 2-ethylhexanoate (Sn(EH)_2_) or ascorbic acid. The reducing agent constantly regenerates an activator, namely Cu(I) species from the Cu(II) compounds. Therefore the polymerization can be initiated using oxidatively stable Cu(II) species instead of Cu(I) compounds [32,33]. Furthermore, as Sn(EH)_2_ is not able to initiate polymerization, the ARGET ATRP should allow synthesis of pure block copolymers, not contaminated by a fraction of homopolymers. Last but not least, the use of a reducing agent in the ARGET ATRP allows removal of residual oxygen from the polymerization system. Thanks to this, the degassing procedure before the polymerization does not have to be so restrictive.

We tested three types of catalytic system with three ligands (L) differing in their activities in ATRP: N,N′,N″,N″-pentamethyldiethylenetriamine (PMDETA), 2,2′-Bipyridyl (bpy) and tris[2-(dimethylamino)ethyl]amine (Me_6_TREN). All polymerizations were carried out using a very low concentration of Cu(II) salt (CuCl_2_ or CuBr_2_) (9 ppm (*w/w*)). In all cases, the same molar ratio of PNIPAM-Br: St:CuX_2_:L:Sn(EH)_2_ was applied 1:400:0.006:0.1:0.1. The reactions were carried out at 90 °C or 110 °C.

The conditions under which the polymerizations of styrene were performed are presented in Table 3. The obtained conversions of styrene and characteristics of the prepared block copolymers, from SEC, are also given in Table 3.

As can be seen, the highest conversion (>50%) was attained for CuCl_2_/Me_6_TREN catalytic system after 24 h of polymerization which was conducted at 110 °C. However, the polymers with a broad MWD (PDI = 2.01) were prepared in this way. The application of the CuBr_2_/bpy system caused the polymerization to proceed significantly slower but MWDs of the synthesized polymers were still not satisfactory. Finally, the employment of CuCl_2_/PMDETA as a catalytic system resulted in relatively low polydispersities (PDI < 1.5)

**Figure 7:**
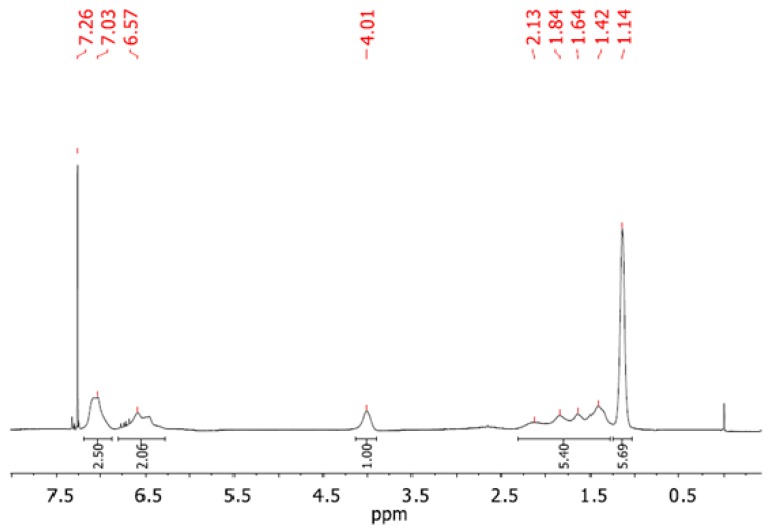
The 300 MHz ^1^H NMR spectrum of diblock copolymer PNIPAM-*b*-PS (recorded for the sample denoted as PNPS2 in Table 3) in CDCl_3_.

Figure 7 shows a ^1^H NMR spectrum recorded for one of the prepared copolymers, denoted in Table 3 as PNPS2. The spectrum reveals the presence of both blocks in the prepared copolymer. From the proton integration of the signals at 6.25 ppm to 7.25 ppm (characteristic for aromatic protons in the PS block and NH proton in the PNIPAM block) and 4.01 ppm for isopropyl proton (CH(CH_3_)_2_) of the PNIPAM block, the ratio of the repeated units in both blocks may be estimated as 1:0.7 PNIPAM:PS. It is in a good agreement with the results from SEC (see Table 3). The ^1^H NMR of other synthesized copolymers can be found in in the Supplementary Material (Figure S2).

It is worth mentioning that in all post-polymerization mixtures, except for the prepared block copolymers, an admixture of polystyrene was present. Albeit the polystyrenes, obtained as by-products, can be easily separated from the prepared copolymers simply by washing the solid mixtures with toluene. Nevertheless, these insights are at odds with the claims that the ARGET ATRP enables synthesis of pure block copolymers uncontaminated by homopolymer fractions [33]. On the one hand, the thermal autopolymerization of styrene can be responsible for the presence of homopolymers in our system.

However, the formation of polystyrene was observed even for relatively short periods of polymerization time (4 h) and at relatively low temperatures (90 °C), which suggests the presence of an initiator for styrene polymerization in the system. We speculate that the paramagnetic Cu(II) organic complexes formed as transition states during the polymerization could play the role of such an initiator under the conditions of ARGET ATRP. We did not observe formation of polystyrene in the case when ATRP (instead of ARGET ATRP) was used for the similar system (for the conditions under which chain extension with styrene via ATRP was performed and characteristic of the synthesized polymers see Table S3 and Figure S5 in the Supplementary Material).

**Figure 8:**
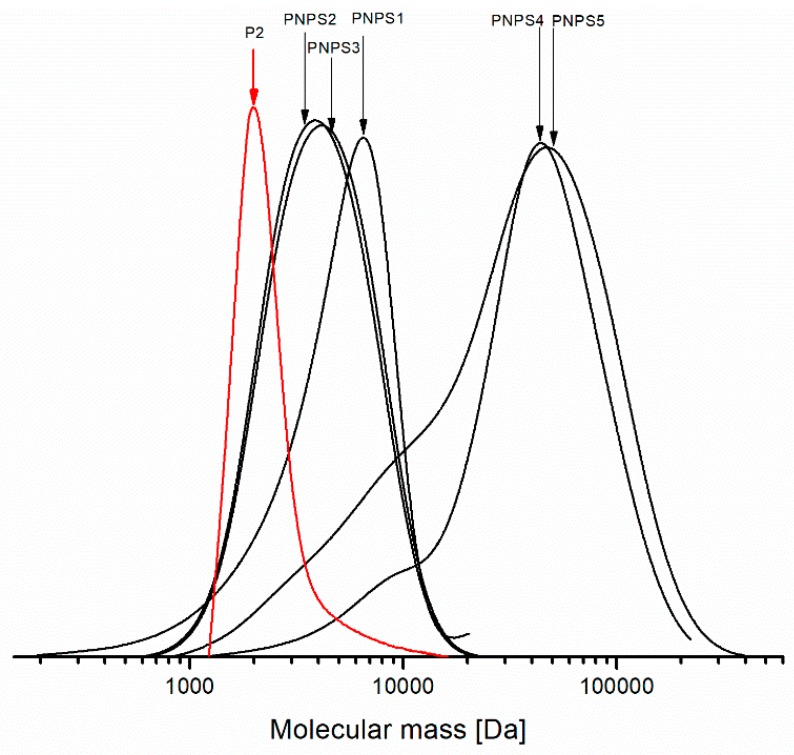
The molecular mass distribution obtained from the SEC analyses for the synthesized PNIPAM-*b*-PS copolymers (see a description of the samples in Table 3) and PNIPAM-Br (denoted in red) used as a macroinitiator for the preparation of PNPS2 and PNPS3.

Figure 8 shows the molecular mass distribution curves determined from the SEC analyses for the synthesized copolymers and for the macroinitiator PNIPAM-Br (denoted in red) used for the preparation PNPS2 and PNPS3 copolymers.

As can be seen in Figure 8, chain extension during the polymerization process is evident. For all the studied copolymers, we observe a monomodal distribution of molecular mass, whereas for PNPS4 and PNPS5 the distribution curves are broad with an accompanying shoulder on the lower masse side. The shoulders visible in the distribution curves are due to the contributions of terminated polymer chain fractions in the polymer sample. On the other hand, the mass distribution curves for PNPS2, PNPS3 are symmetric without the shoulders and the distribution of the molecular mass is relatively narrow for both of them. For the sample denoted as PNPS1, the distribution is narrow, albeit with a small tailing of the peak occurring. It is a likely result of the presence a small fraction composed of fast growing and terminated polymer chains. In general, the SEC analyses proved that the narrowest MWD (among those systems studied) could be achieved using CuCl_2_/PMDETA as a catalytic system via ARGET ATRP procedure.

Additionally, decreasing the temperature from 110 °C to 90 °C causes a further decrease in PDI value of the synthesized polymers.

Taking into account a degree of functionalization of PNIPAMs with bromine (75–85%), we can expect about 15–25% inactive homopolymer molecules in the chain extension with styrene. Thus, most likely, the copolymer samples contain an admixture of PNIPAM. This could explain the presence of the low molecular weight fractions, which are visible in the SEC curves in Figure 8. In our opinion preparative size-exclusion chromatography could be used for the purification of copolymers from residual amount of PNIPAMs.

**Figure 9:**
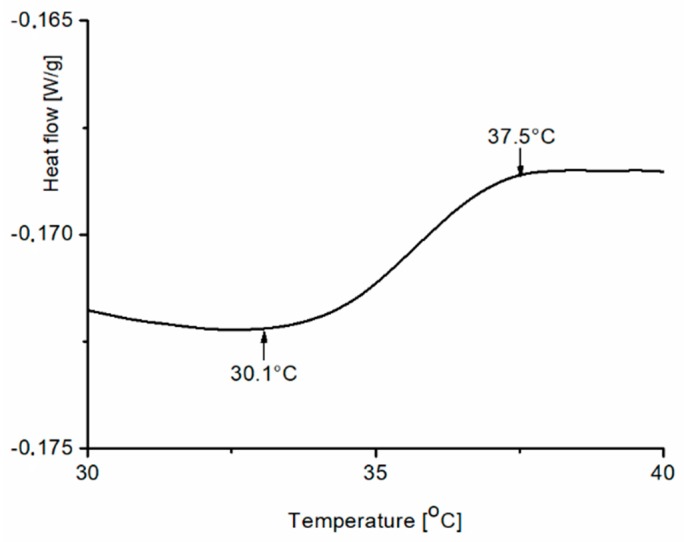
The heating DSC thermogram for an aqueous solution of PNPS2 (0.5% *w/w*), heating rate of 1 K/min.

The synthesized block copolymers were analyzed to check their thermosensitivities using DSC and DLS. The aqueous solutions of the prepared copolymers were studied by DSC within the range of temperatures 30–45 °C. Figure 9 shows an example of a heating DSC thermogram for one sample among the synthesized copolymers (PNPS2) with a heat capacity change observed between 30.1 °C and 37.5 °C.

There is no a typical endothermic DSC peak in the thermogram, only a jump of the line within the expected range of temperatures. We expected such a range of temperature since a decrease in LCST after introducing a hydrophobic block into the PNIPAM chain was reported earlier [34] as the phase transition of polymers, used as macroinitiator in styrene polymerization, occurs between 42.8 °C and 44.3 °C. Kali et al. [35] also observed a broad thermal transition for amphiphilic conetworks consisting PNIPAM cross-linked by hydrophobic polymer. They also observed that the decrease of LCST is proportional with hydrophobic polymer content.

DLS also confirmed the thermosensitive properties of the synthesized copolymers in aqueous solutions. We observed that the sizes of particles detected in aqueous solution of copolymers decrease as a result of heating. For instance, in an aqueous solution of copolymer PNPS2 the determined maximum of number-averaged hydrodynamic diameter of particles was 600 nm at 25 °C; whereas at 45 °C this maximum is visible at 300 nm (see Figure S4 in the Supplementary Material). The observed decreases of particles sizes can be explained by the shrinking of PNIPAM blocks in the polymer chains under the influence of heating above their LCSTs. Such a phenomenon (aggregate contraction as the temperature is increased above LCST) was reported for thermoresponsive block copolymers [36].

**Figure 10:**
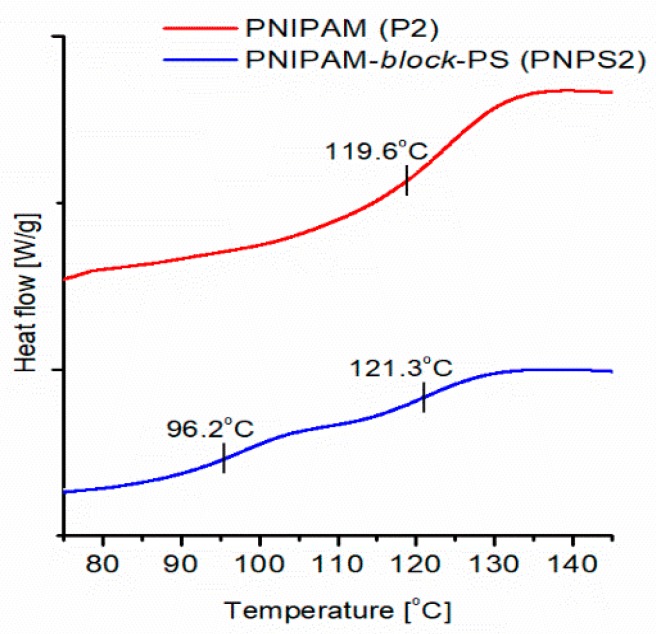
The DSC thermograms for the synthesized homopolymer, copolymer and *T*_g_s determined from the thermograms (cooling scan, cooling rate of 20 K/min).

DSC measurements, conducted for solid dry copolymer samples, allowed corroboration of the presence of two chemically different block phases separated in the molecules of the prepared copolymers. Figure 10 shows the DSC thermogram recorded for an exemplar copolymer sample (PNPS2). The glass transition temperatures (*T*_g_) determined from this curve are shown in Figure 10.

In the thermogram of the synthesized copolymer, two separate *T*_g_s are observed at 96.2 °C and 121.3 °C corresponding to the PS and PNIPAM blocks, respectively.

The presence of two separate *T*_g_s in the thermogram is direct proof that there are separated homopolymer blocks in the synthesized copolymer chains. This is because, as is well-known, a mixture of miscible homopolymers or random copolymers exhibits only one averaged *T*_g_ [37,38,39]. For all synthesized copolymers, DSC thermograms showed two separate *T*_g_s. This demonstrates that the developed procedure indeed leads to copolymers with separated homopolymer blocks.

## 4. Conclusions

In summary, we have presented a novel strategy for the synthesis of block amphiphilic and thermosensitive copolymers with the sequence PNIPAM-*b*-PS. The proposed synthetic route consists of four stages and is based on the application of 2-chloro-N-(2-hydroxyethyl)propanamide (NCPAE) as bifunctional initiator. First, NCPAE was used in the ATRP of N-isopropylacrylamide (NIPAM) and hydroxyl functionalized PNIPAMs with low polydispersities (1.1 ≤ PDI ≤ 1.3) and molecular masses in the range 2,000–12,000 Da were synthesized. Next, terminal chlorines were removed from the prepared PNIPAM molecules by a dehalogenation process, followed by polymer chain modification using α-bromoisobutyryl bromide. The obtained modified polymers (PNIPAM-Br) were used as macroinitiators in ARGET ATRP of styrene. The developed procedure gave well-defined block copolymers, with two chemically different blocks phase separated in the molecules. We proved that the use of CuCl_2_/PMDETA as catalytic system at concentration as low as 9 ppm, under the conditions of ARGET ATRP, results in copolymers with relatively low polydispersities (PDI ≈ 1.3). To achieve narrower MWDs of the prepared copolymers, further improvement of the proposed procedure is necessary. We think that the slowing down of NIPAM’s polymerization and increasing of degree of PNIPAMs’ functionalization with bromine should be crucial factors. Further experiments are in progress.

Both the DSC and DLS techniques verified thermosensitive behavior of the synthesized PNIPAMs and PNIPAM-*b*-PS copolymers generated from them.

The proposed procedure can be applied not only for the preparation of PNIPAM-*b*-PS copolymers, but can also be used for the synthesis of other block copolymers that are built of monomers with different activities in ATRP. Changing the terminal halogen atoms is necessary to achieve good control over the polymer chain extension.

Due to their amphiphilic character, thermosensitive behavior and biocompatibility, the synthesized copolymers may find numerous interesting applications e.g., drug delivery systems, tissue engineering, regenerative medicine, construction of smart membranes.

## Figures and Tables

**Table 1:** The molar ratios applied in the polymerization of N-isopropylacrylamid (NIPAM), monomer conversions, number-averaged molar masses (*M*_n_) and polydispersity indices from Size-Exclusion Chromatography (SEC) analyses. All polymerizations were carried out in isopropanol (NIPAM/isopropanol = 1:2 *w/w*) at room temperature.

Sample	Time [h]	[I]/[M]/[CuCl]/[L] ^a^	Conv ^b^ [%]	*M*_n_ [Da]	Theoretical ^d^ *M*_n_ [Da]	PDI (*M*_w_/*M*_n_)
P1	3	1:50:1:1	35.2	2150 ^c^	1990	1.14 ^c^
P2	4	1:50:1:1	40.2	2200 ^c^	2270	1.17 ^e^
P3	5	1:50:1:1	61.0	3300 ^c^	3050	1.13 ^c^
P4	22	1:50:1:1	87.2	4100 ^c^	4900	1.14 ^c^
P5	23	1:50:1:1	89.2	4900	5050	1.11 ^c^
P6	2	1:200:1:1	63.8	11700 ^c^	14400	1.31 ^c^

^a^ I = NCPAE, M = NIPAM, L = Me_6_TREN; ^b^ as determined gravimetrically; ^c^ from SEC in 1% (*w/v*) LiBr/DMF; ^d^ calculated as *M*_n_ = conv × ([M]/[I]) × M_NIPAM_; ^e^ determined from SEC in THF.

**Table 2:** Results of Differential Scanning Calorymetry (DSC) measurements of aqueous solutions of homopolymers (1.5% *w/w*). The temperatures given below corresponds to: *t*_o_—onset of the peak, *t*_p_—minimum of the peak and *t*_r_— range of temperatures within the transition occurs. The phase transition enthalpies ΔH have been estimated as the areas of the endothermic peaks corresponding to the phase transitions.

Sample	*t*_o_ [°C]	*t*_p_ [°C]	*t*_r_ [°C]	Δ*H* [J/g] ^a^
P1	42.8	43.0	42.8 ÷44.6	14.4
P2	42.8	42.9	42.8 ÷ 44.3	14.3
P3	43.1	43.8	43.1 ÷ 45.1	2.98
P4	40.7	41.1	40.7 ÷ 43.0	6.43
P5	43.4	43.4	43.4 ÷ 44.7	8.06
P6	40.7	40.6	40.7 ÷ 42.4	6.74

^a^ determined as endothermic peak area calculated per 1 g of solution (concentration of all solutions 1.5% (*w/w*)).

**Table 3:** The conditions under which the chain extensions of PNIPAM-Br with styrene via ARGET ATRP were conducted; the obtained monomer conversions (Conv), number-averaged molecular masses (*M*_n_) of both blocks in the polymers’ chains and polydispersity indices determined from SEC analyses.

Entry	Starting PNIPAM	Catalytic System	*t* [h]	*T* [°C]	Conv ^a^ [%]	*M*_n_ [Da] ^b^ PNIPAM	*M*_n_ [Da] ^b^ PS	PDI = *M*_w_/*M*_n_
PNPS1	P5	CuCl_2_/PMDETA	46	110	37.5	4900	2600	1.33
PNPS2	P2	CuCl_2_/PMDETA	4	90	10	2200	3000	1.35
PNPS3	P2	CuCl_2_/PMDETA	8	90	28.4	2200	4300	1.35
PNPS4	P4	CuBr_2_/bpy	48	110	38.7	4100	11100	1.7
PNPS5	P5	CuCl_2_/Me_6_TREN	24	110	54.8	4900	19500	2.01

^a^ determined from GC analysis; ^b^ from SEC in 1% (*w/v*) LiBr/DMF (molecular mass of polystyrene (PS) block as the second was calculated as the difference between the molecular mass of the copolymer and PNIPAM-Br used as an initiator in the polymerization of styrene).

## Data Availability

The raw/processed data is available upon reasonable request.

## Supplementary Materials

The following are available online at https://www.mdpi.com/2073-4360/11/9/1484/s1. The number averaged hydrodynamic diameter distribution in aqueous solutions (1.5% *w/w*) of homopolymers (descrption of the samples in Table 1 in main document) at 25, 35 and 40 °C from DLS measurements. For P5 the changes in the size of particles at 35 °C have not be observed in comparison with the sizes which observed at 25 °C; The conditions under which the chain extensions of PNIPAM-Cl with styrene via ARGET ATRP were conducted; number-averaged molecular masses (*M*_n_) of copolymers and macroinitiator determined from SEC analyses; estimated number of styrene mers in copolymers’ chains; Results of SEC analyses (with THF as eluent) for the prepared homopolymers before and after removal of terminal chlorine atoms as well as after esterification by using α-bromoisobutyryl bromide; The 300 MHz ^1^H NMR spectrum of diblock copolymer PNIPAM-b-PS (recorded for the sample denoted as PNPS3 in Table 3) in CDCl_3_; The photo of aqueous solution of the synthesized diblock copolymer PNIPAM-*b*-PS (a solution of sample PNPS3). As the molecule of PNPS3 is amphiphilic characteristic foam appears as a result of surface tension decreasing; The conditions under which of the chain extensions of PNIPAM-Br with styrene via ATRP were conducted; number-averaged molecular masses (*M*_n_) of copolymers and macroinitiator determined from SEC analyses; ^1^H NMR spectrum of copolymer PNPS(200)21 synthesized via ATRP under conditions reported in Table S3.
