# Supplementary material for: A Novel Strategy for the Synthesis of Amphiphilic and Thermoresponsive Poly(N-isopropylacrylamide)-b-Polystyrene Block Copolymers via ATRP
Source: Polymers (Basel). 2019 Sep 11;11(9):1484. doi: 10.3390/polym11091484 (PMC6780390; doi:10.3390/polym11091484)
Supplement: Supplementary file 1 [file polymers-11-01484-s001.pdf]

polymers

MDPI

Supplementary Material

Table S1. The conditions under which the chain extensions of PNIPAM-Cl with styrene via ARGET ATRP were conducted; number-averaged molecular masses (M_n) of copolymers and macroinitiator determined from SEC analyses; estimated number of styrene mers in copolymers' chains.

Sample	Time [h]	[I]/[M]/[$CuCl_2$]/[L]/[Sn(EH)$_2$] [a]	M_n PNIPA M^b [Da]	M_n copolymer[b] [Da]	Estimated number of mers[c]
PNPSI	23	1 : 200 : 0.002 : 0.1 : 0.4	4100	4700	6
PNPSII	24	1 : 200 : 0.005 : 0.1 : 0.48	4100	4200	1
PNPSIII	93	1 : 200 : 0.005 : 0.85 : 0.08	4100	4400	3
PNPSIV	79	1 : 200 : 0.03 : 0.08 : 0.15	3300	3900	6
PNPSV	54	1 : 200 : 0.3 : 0.3 : 0.3	3300	4100	8
PNPSVI	64	1 : 200 : 0.3 : 0.3 : 0.5	3900	4600	7
PNPSVII	96	1 : 200 : 0.3 : 0.3 : 1.5	3500	3700	2

[a] I = PNIPAM-Cl molecules terminated by chlorine (macroinitiator), M = styrene (monomer), L = Me_6TREN (ligand); [b] from SEC in 1% (w/v) LiBr/DMF, [c] calculated as the difference between number-average molecular masses of copolymer and PNIPAM-Cl used in the chain extension.

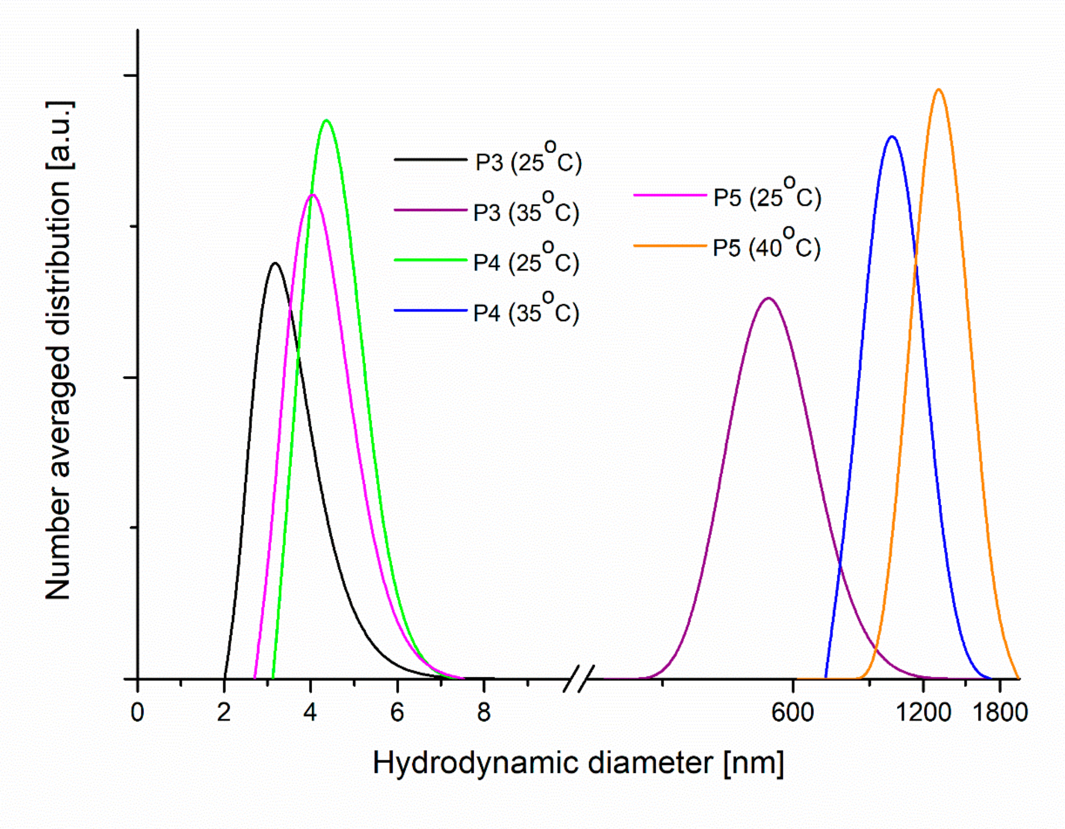

Figure S1. The number averaged hydrodynamic diameter distribution in aqueous solutions (1.5% *w/w*) of homopolymers (descrption of the samples in Table 1 in main document) at 25, 35 and 40 ^{0}C from DLS measurements. For P5 the changes in the size of particles at 35°C have not be observed in comparison with the sizes which observed at 25°C.

Table S2. Results of SEC analyses (with THF as eluent) for the prepared homopolymers before and after removal of terminal chlorine atoms as well as after esterification by using α-bromoisobutyryl bromide.

**PNIPAM-Cl**		**After Dehalogenation (PNIPAM-H)**		**After Esterification (PNIPAM-Br)**	
**M_n [Da]**	**PDI**	**M_n [Da]**	**PDI**	**M_n [Da]**	**PDI**
1500	1.53	2000	1.48	2200	1.49
3400	1.28	4000	1.15	4000	1.15
4100	1.41	5200	1.34	5400	1.30
5600	1.36	6100	1.30	6000	1.30

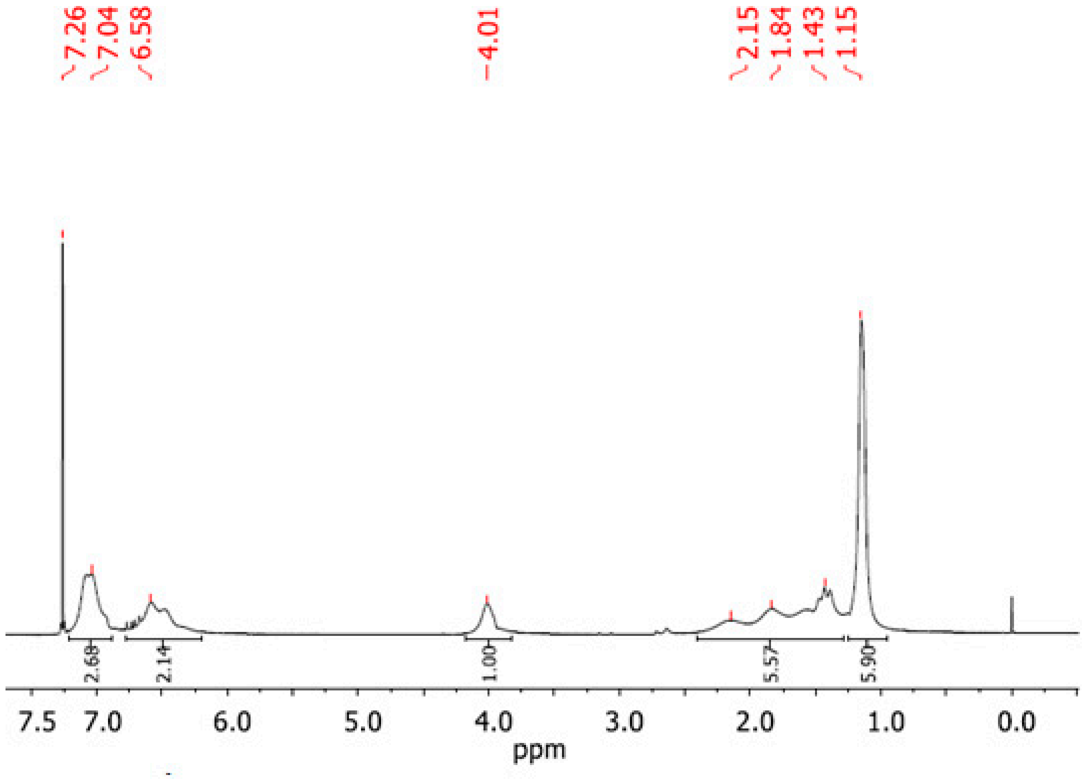

Figure S2. The 300 MHz ^{1}H NMR spectrum of diblock copolymer PNIPAM-*b*-PS (recorded for the sample denoted as PNPS3 in Table 3) in $CDCl_3$.

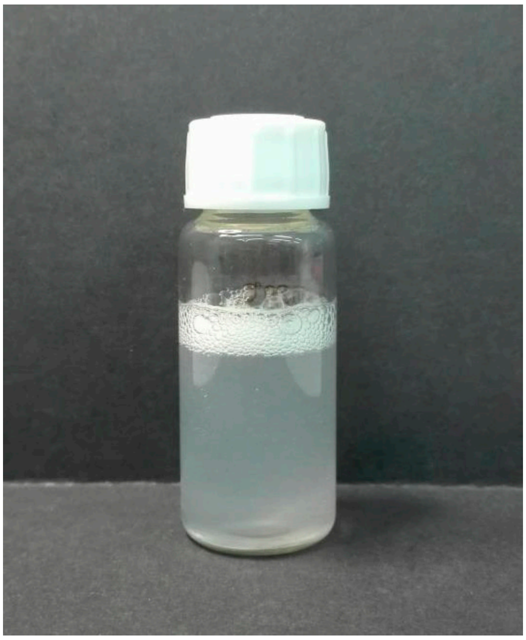

Figure S3. The photo of aqueous solution of the synthesized diblock copolymer PNIPAM-*b*-PS (a solution of sample PNPS3). As the molecule of PNPS3 is amphiphilic characteristic foam appears as a result of surface tension decreasing.

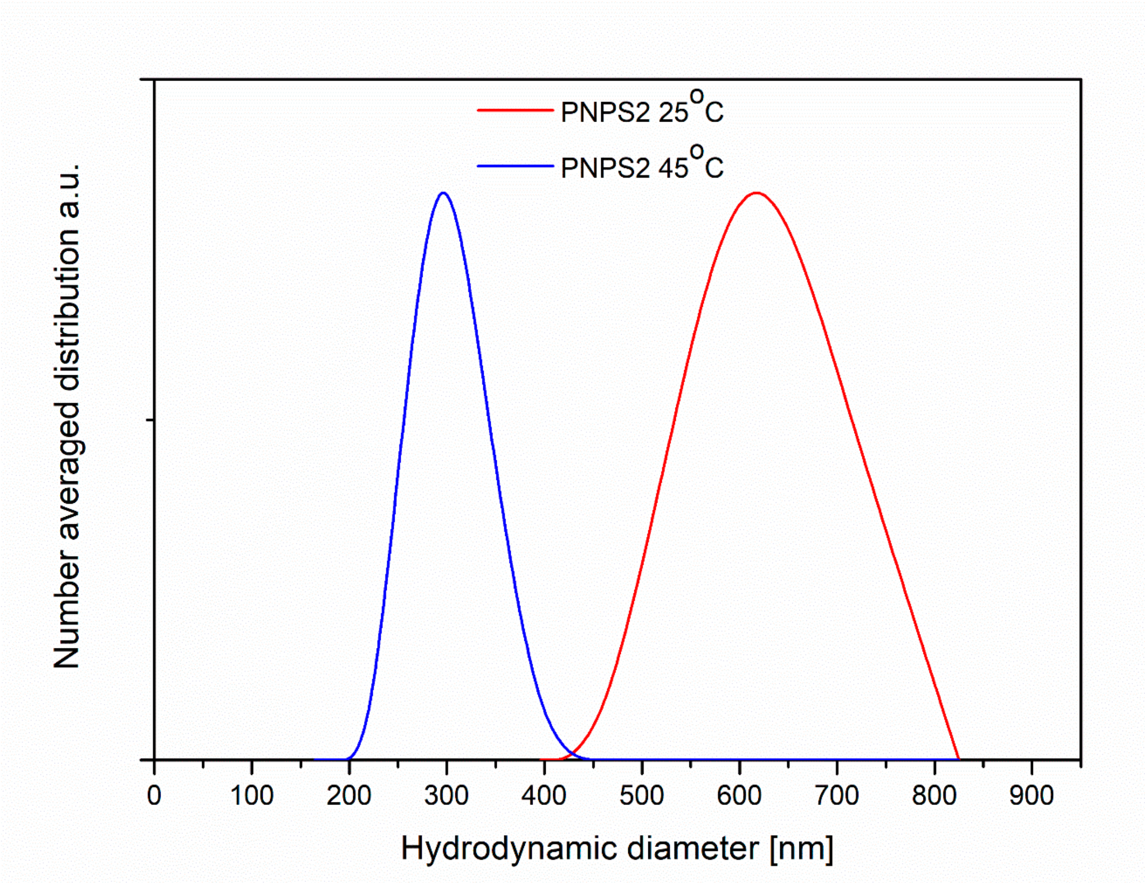

Figure S4. The number averaged hydrodynamic diameter distribution in aqueous solutions of PNPS2 at 25 and 45°C from DLS measurements.

Table S3. conditions under which of the chain extensions of PNIPAM-Br with styrene *via* ATRP were conducted; number-averaged molecular masses (M_n) of copolymers and macroinitiator determined from SEC analyses.

Sample	Time [h]	Temp. [°C]	[I]/[M]/[CuBr]/[L][a]	M_n PNIPAM[b] [Da]	M_n copolymer[b] [Da]
PNPS(200)21	21	90	1 : 200 : 2 : 2	11753	17950

[a] I = PNIPAM-Br (macroinitiator), M = styrene (monomer), L = bpy (ligand); [b] from SEC in 1% (w/v) LiBr/DMF.

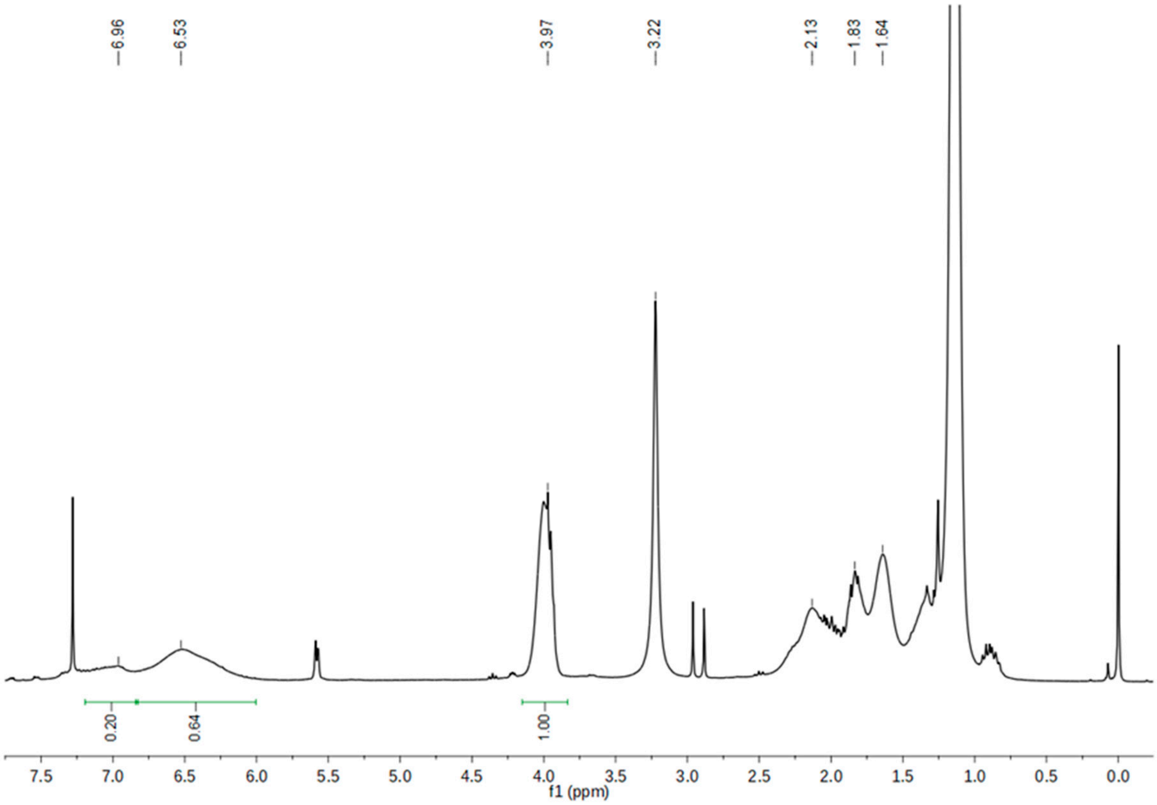

Figure S5. ^{1}H NMR spectrum of copolymer PNPS(200)21 synthesized via ATRP under conditions reported in Table S3.
